# Revealing Genome-Based Biosynthetic Potential of *Streptomyces* sp. BR123 Isolated from Sunflower Rhizosphere with Broad Spectrum Antimicrobial Activity

**DOI:** 10.3390/antibiotics11081057

**Published:** 2022-08-04

**Authors:** Neelma Ashraf, Sana Zafar, Roman Makitrynskyy, Andreas Bechthold, Dieter Spiteller, Lijiang Song, Munir Ahmad Anwar, Andriy Luzhetskyy, Ali Nisar Khan, Kalsoom Akhtar, Shazia Khaliq

**Affiliations:** 1 Industrial Biotechnology Division, National Institute for Biotechnology and Genetic Engineering (NIBGE), Constituent College of Pakistan Institute of Engineering and Applied Sciences (PIEAS), Jhang Road, PO Box 577, Faisalabad 38000, Pakistan; 2 Department of Chemical Ecology/Biological Chemistry, University of Konstanz, 78457 Konstanz, Germany; 3 Department of Pharmaceutical Biology and Biotechnology, Institute of Pharmaceutical Sciences, University of Freiburg, 79104 Freiburg im Breisgau, Germany; 4 Department of Chemistry, University of Warwick Coventry, Coventry CV4 7AL, UK; 5 Pharmaceutical Biotechnology Campus, Saarland University, Building C2.3, 66123 Saarbrucken, Germany

**Keywords:** *Streptomyces*, secondary metabolites, genome, biosynthetic gene clusters, high-performance liquid chromatography (HPLC), mass spectrometry

## Abstract

Actinomycetes, most notably the genus *Streptomyces*, have great importance due to their role in the discovery of new natural products, especially for finding antimicrobial secondary metabolites that are useful in the medicinal science and biotechnology industries. In the current study, a genome-based evaluation of *Streptomyces* sp. isolate BR123 was analyzed to determine its biosynthetic potential, based on its in vitro antimicrobial activity against a broad range of microbial pathogens, including gram-positive and gram-negative bacteria and fungi. A draft genome sequence of 8.15 Mb of *Streptomyces* sp. isolate BR123 was attained, containing a GC content of 72.63% and 8103 protein coding genes. Many antimicrobial, antiparasitic, and anticancerous compounds were detected by the presence of multiple biosynthetic gene clusters, which was predicted by in silico analysis. A novel metabolite with a molecular mass of 1271.7773 in positive ion mode was detected through a high-performance liquid chromatography linked with mass spectrometry (HPLC-MS) analysis. In addition, another compound, meridamycin, was also identified through a HPLC-MS analysis. The current study reveals the biosynthetic potential of *Streptomyces* sp. isolate BR123, with respect to the synthesis of bioactive secondary metabolites through genomic and spectrometric analysis. Moreover, the comparative genome study compared the isolate BR123 with other *Streptomyces* strains, which may expand the knowledge concerning the mechanism involved in novel antimicrobial metabolite synthesis.

## 1. Introduction

The growing resistance of pathogenic microorganisms to antimicrobial agents has become a global problem [1]. There is a dire need to discover newer antibiotics and techniques that can overcome this problem [2,3]. In the development of new therapeutical agents, natural products play a vital role. More than 2200 biologically active compounds have been isolated from naturally abundant microorganisms [4,5]. Many novel antibiotics were discovered from soil bacteria as well as from marine habitats.

Actinomycetes are a group of aerobic, gram-positive, sporulating, and filamentous bacteria that have aerial and substrate mycelium, with the ability to produce many bioactive secondary metabolites [6]. Among the class Actinobacteria, the genus *Streptomyces*, primarily found in the soil and aquatic habitats, has gained much attention because of its role in the production of novel antimicrobial metabolites. More than 7630 bioactive compounds have been reported to be only produced by this genus [7]. These bioactive compounds are the result of an unprecedented genetic potential through biosynthetic gene clusters (BGCs), which are harbored in their genomes and contain genes arranged in close vicinity. The BGCs are under the control of a sophisticated regulatory network and the laboratory conditions used [8]. Hence, the same species isolated from different habitats can have different sets of biosynthetic gene clusters, which may be lost or gained when a particular strain is transferred to a new environment [9]. Biosynthetic gene clusters (BGCs) have been classified into two main pathways based on their products, i.e., nonribosomal peptide synthetases (NRPSs) and polyketide synthases (PKSs), for the biosynthesis of potent secondary metabolites. Polyketide synthases (PKSs) are further divided into PKS-I and PKS-II gene clusters, where the diversity evolution of PKSs can be achieved by using fragments of genes PKS-I ketosynthase and PKS-II KSα domains. Conversely, NRPSs are produced by nonribosomal peptide synthase (NRPS) gene clusters and to achieve their diversity evolution, their adenylation (AD) domains are used. Both the NRPS and PKS products are comprised of remarkably long genes (>5 kb) that encode multi-modular enzymes with repetitive domain structures. In addition, other well-known classes of BGCs are terpenoids, saccharides and lanthipeptides [10,11]. 

The conventional approach to discovering antibiotics from *Streptomyces* is through the bioactivity-based identification of a compound, using mass spectrometry and nuclear magnetic resonance (NMR) analyses [12]. However, the genome-based approaches have divulged that most of the BGCs are not expressed under certain laboratory conditions, proposing that the capability of *Streptomyces* to produce secondary compounds has been underestimated [13,14]. On average, each *Streptomyces* has the potential to produce more than 30 secondary metabolites, meaning that they are a valuable source of natural product discovery [15]. The genomic data of over 1141 strains of *Streptomyces* are deposited and available in the GenBank database. In this study, we conducted a detailed analysis of *Streptomyces* sp. BR123, which was isolated from the rhizosphere of a sunflower plant. The analysis was based on its in vitro antimicrobial activities in relation to the whole genome sequencing data and a general comparison with other reported strains of the genus *Streptomyces*.

## 2. Materials and Methods

### 2.1. Isolation and Cultivation Conditions of Streptomyces sp. BR123

Soil samples were collected from the rhizosphere of sunflower plants located in various agricultural fields of Faisalabad, Pakistan for the purpose of isolating *Streptomyces* colonies. From each sample, 1 g of dried soil was added into 9 mL of double distilled autoclaved water and mixed well. The diluted aliquots (0.1 mL), 10^−1^, 10^−2^, 10^−3^, 10^−4^, and 10^−5^ were spread into petri plates containing a starch casein agar (SCA) medium, composed of: soluble starch 10.0 g, KNO_3_ 2.0 g, casein 0.3 g, K_2_HPO_4_ 2.0 g, NaCl 2.0 g, MgSO_4_∙7H_2_O 0.05 g, FeSO_4_∙7H_2_O 0.01 g, CaCO_3_ 0.02, agar 20 g, and distilled water 1 L [16]. The pH of the medium was adjusted to be 7.0–7.2. The medium was supplemented with an antifungal solution of cycloheximide (100 µg/mL) to inhibit fungus growth, and plates were incubated at 30 °C for 5–7 days. Colonies that showed hard texture and filamentous mycelium when observed under a phase contrast microscope were picked and purified by using an agar streak method [17]. The purified stock cultures were preserved in glycerol (40% *v*/*v*) at −80 °C. Moreover, *Streptomyces* sp. BR123 was cultivated in a starch casein broth at 30 °C, rotated at 180 rpm for 7 days for later analysis. 

### 2.2. Sequencing and Assembly of the Genome

To perform the genome-based comparative analysis, the biosynthetic potential of *Streptomyces* isolate BR123 was investigated at the level of draft genome sequence. The biomass of the isolate BR123 was separated from the liquid culture and grown for 72 h at 30 °C in casein-starch-peptone-yeast extract-malt extract (CSPY-ME) broth with the composition (in g/L): K_2_HPO_4_ 0.5, starch 10, casein 3, yeast extract 1, malt extract 10, and peptone 1. The broth’s final pH was 7.2. Genomic DNA of high quality was obtained through the bead method and quantification was performed by a high-sensitivity (HS) assay of Quant-iT double-stranded DNA (dsDNA) (ThermoFisher Scientific, Waltham, MA, USA). The genomic DNA was sequenced at MicrobesNG using the Nextera XT Library Preparation Kit (Illumina, San Diego, CA, USA). For the generation and quantification of the Illumina library, the KAPA Biosystems Library Quantification Kit was used. The genomic data were deposited at the National Centre for Biotechnology Information (NCBI) under the accession number PRJNA643667. Trimmomatic 0.30 was used to compile raw reads, with a quality cutoff of Q15 [18].

### 2.3. Annotation of Genome and Bioinformatics Analysis

For the annotation of the genome, Rapid Annotation using Subsystem Technology (RAST) version 2.0 was used [19]. For the assembly of matrices, PGAP (Prokaryotic Genome Annotation Pipeline) v4.2 from the NCBI was used. The predictions of gene clusters with the potential to produce secondary metabolites were analyzed by using the online antiSMASH (antibiotics & Secondary Metabolite Analysis Shell) bacterial version, accessed on 22 April 2022.

### 2.4. Amplification of NRPS and PKS Genes by PCR 

The PKS-I, PKS-II, and NRPS genes were amplified using the following primer sets, K1F (5′-TSAAGTCSAACATCCGBCA-3′)/M6R (5′-CGCAGGTTSCSGTACCAG TA-3′) [20], KSα (5′-TSGCSTGCTTGGAYGCSATC-3′)/KSβ (5′-TGGAANCCGCCGAABCCGCT-3′), and A3F (5′-GCSTACSYSATSTACACSTCSGG-3′)/A7R (5′-SASGTCVCCSGTSGCGTA S-3′). The reaction for NRPS and PKS genes was made with the final volume of 50 µL containing 1.5 µL of extracted genomic DNA, 1 µL of each primer (10 pmol), 21.5 µL of nuclease-free water, and 25 µL of dream taq (PCR master mix). The amplification process was performed in Analytik Jena Flex Thermal cycler block assembly 96 G, according to the following specified conditions for each primer: 5 minutes at 95 °C for denaturation and 35 cycles of 30 seconds at 95 °C; 2 minutes at 57 °C, 63 °C, and 59.7 °C for K1F/M6R, KSα/KSβ, and A3F/A7R, respectively; 4 minutes at 72 °C; and 10 minutes at 72 °C. Gel electrophoresis was used to analyze the PCR products using 1% agarose gel final stained with ethidium bromide and the end product was purified with the help of GeneJET PCR Purification Kit K0721 (Thermo scientific/Vilnius, Lithuania).

### 2.5. Assessment of Antimicrobial Potential

The isolate BR123 was checked for antimicrobial potential through the agar-well diffusion method [21] against 2 gram-positive bacteria (*Staphylococcus aureus* and *Bacillus subitilis*), 4 gram-negative bacteria (*Salmonella typhi*, *Xanthomonas oryzae*, *Escherichia coli* and *Pseudomonas aeruginosa*), and 4 fungi (*Aspergillus flavus*, *Aspergillus niger*, *Fusarium solani* and *Fusarium oxysporum*) by using 7 different media (Supplementary Table S1). Plates were overlaid with the test culture and wells were filled with the supernatant of BR123. These plates were incubated for 24 h at 30 °C in case of bacteria and for 5–7 days in the case of fungal for the examination of clear zones formation. 

### 2.6. Analysis of Metabolites through HPLC-MS from Streptomyces sp. BR123

#### 2.6.1. Sample Preparation

*Streptomyces* sp. BR123 was pre-cultivated in a starch casein (SC) broth (pH 7.2). After cultivating for 4 days in a rotary shaker at 180 rpm and 28 °C, 5 mL of the culture was used to inoculate 1 L of casein-starch-peptone-yeast extract-malt extract (CSPY-ME) broth in a 2.8 L flask [17]. Twice extraction of the entire culture was performed with an equal volume of ethyl acetate (EtOAc) by adjusting the pH of the broth to 3.5. To obtain solid material, the ethyl acetate extract was concentrated in a rotary evaporator. 

#### 2.6.2. Analysis of Metabolites

Low resolution electrospray ionization source mass spectra were recorded using a UHPLC focused Thermo Scientific Dionex UltiMate 3000 auto-sampler (Dionex, Thermo Fisher Scientific, Freiburg, Germany), coupled with a TSQ Quantum Access MAX diode array detector (DAD, Thermo Fisher Scientific, Germany). The diode array detector allows for the relative qualification of non-volatile components. Using a mobile phase of water (A) and acetonitrile (B) both containing 0.5% acetic acid, the separation of compounds was performed on a C18 HPLC column (Waters, 3.5 m, 4.6 100 mm). The gradient started by washing for the following durations and concentrations: 0.5 min in 95% A; 19.5 min in 5% A; 23.5 min in 5% A; 24 min in 95% A; 27 min in 95% A; followed by a final washing in 95% A and 5% B solution for 5 min. The column was re-equilibrated. The method lasted a total of 27 min. The flow rate was 0.5 mL/min, column temperature was 30 ± 10 °C, and pressure was adjusted from 5 × 10^2^ to 4 × 10^4^ kPa. Further analysis of the compounds was determined using high resolution Bruker MaXis II Q-TOF (Bruker, Warwick, UK) mass spectrometer coupled with a Dionex 3000RS UHPLC (Bruker, Warwick, UK). The analysis was performed by keeping a mass range of 50–3000 *m*/*z* and using a mobile phase of water (A) and acetonitrile (B), both containing 0.1% formic acid. Separation was again performed by C18 HPLC column. The gradient for the high resolution started from 5% to 100% in 25 min, keeping a flow rate of 0.2 mL/min. The column was washed and re-equilibrated. Mass spectra were recorded in both negative and positive modes and Xcalibur version 4.3 was used for the data analysis.

### 2.7. Comparative Genome Analysis

The complete 16S rRNA sequence data from the genome of all strains were retrieved from TrueBacTMIDBeta [19]. Alignment of the extracted 16S rRNA sequences was achieved through the ClustalW tool available in MEGA Software version 7 [22] and the phylogenetic tree was constructed using the neighbor-joining method with a bootstrap value of 1000. Additionally, the whole genome phylogeny was determined by using the online available version of KBase software. The average nucleotide identity scores were calculated using the FastANI algorithm [23]. 

### 2.8. Accession Number of Genome Sequence

The genome sequence of *Streptomyces* sp. BR123 has been submitted to GenBank under the bio project number PRJNA643667, genome sequencing project number JACBGN000000000, and SRA number SRR12527047. Moreover, the 16S rRNA gene sequence has been submitted to GenBank under the accession number MT799988.

## 3. Results and Discussion

### 3.1. General Genomic Characteristics and Phylogenetic Analysis of Streptomyces sp. BR123

**Figure 1:**
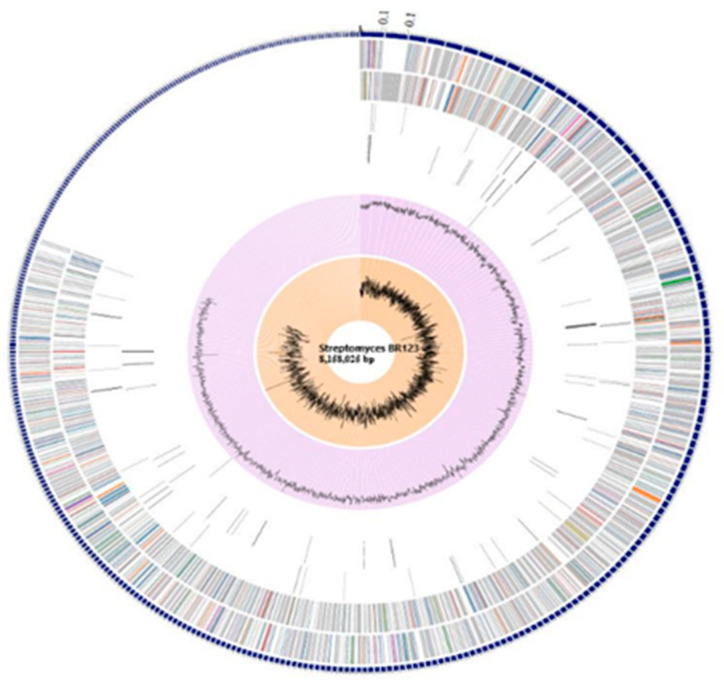
Circular map of the *Streptomyces* isolate BR123 genome, retrieved from PATRIC version 3.6.9. Description of each circle is given from outside in: CDS on the forward strand, CDS on the reverse strand, RNA genes, CDS with homology to known antimicrobial resistance genes, CDS with homology to known virulence factors, GC content, and GC skew.

A genomic sequence with a total stretch of 8,158,025 bp was obtained, and the length of the shortest contig at value N50 was observed to be 22,797 (Figure 1).

An average GC content of 72.63% was observed in the isolate BR123, which is close to that of previously reported *Streptomyces* strains [24,25,26]. A total of 8103 protein coding sequences (CDS), 281 pseudo genes, 8 rRNA genes, and 68 tRNA genes were predicted through Rapid Annotation using Subsystem Technology (RAST) [27,28]. Table 1 provides the genomic characteristics of *Streptomyces* sp. BR123 in comparison to certain other available genomes of *Streptomyces* strains. 

**Figure 2:**
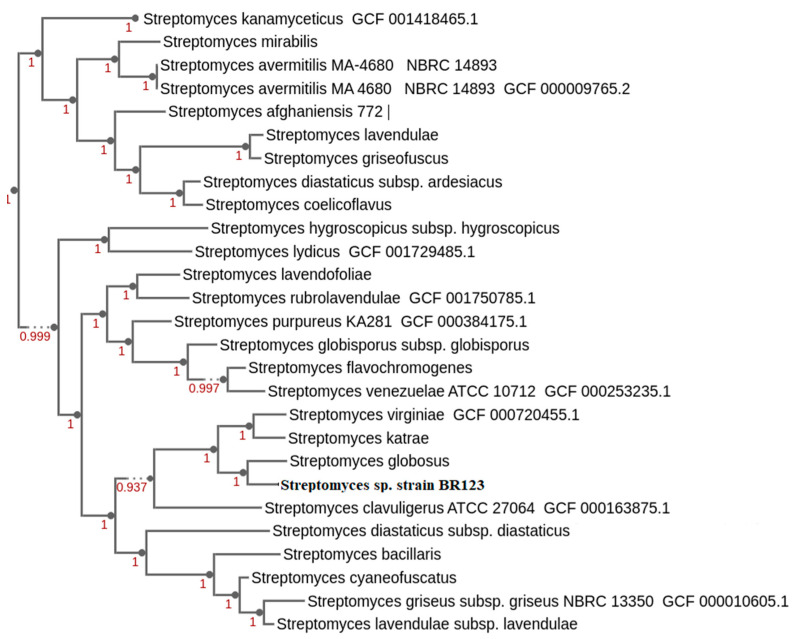
Whole genome-based tree of *Streptomyces* isolate BR123 with other *Streptomyces* strains, inferred using Kbase.

The taxonomic position of the *Streptomyces* sp. BR123 was determined within the genus *Streptomyces* (Supplementary Figure S2). Additional confirmation of this was performed by a genome-based phylogenetic analysis of the isolate BR123 in comparison with other *Streptomyces* strains [29,30]. *Streptomyces* sp. BR123 was closely branched with three other *Streptomyces* species and most closely branched with *Streptomyces globosus* (Figure 2). 

**Figure 3:**
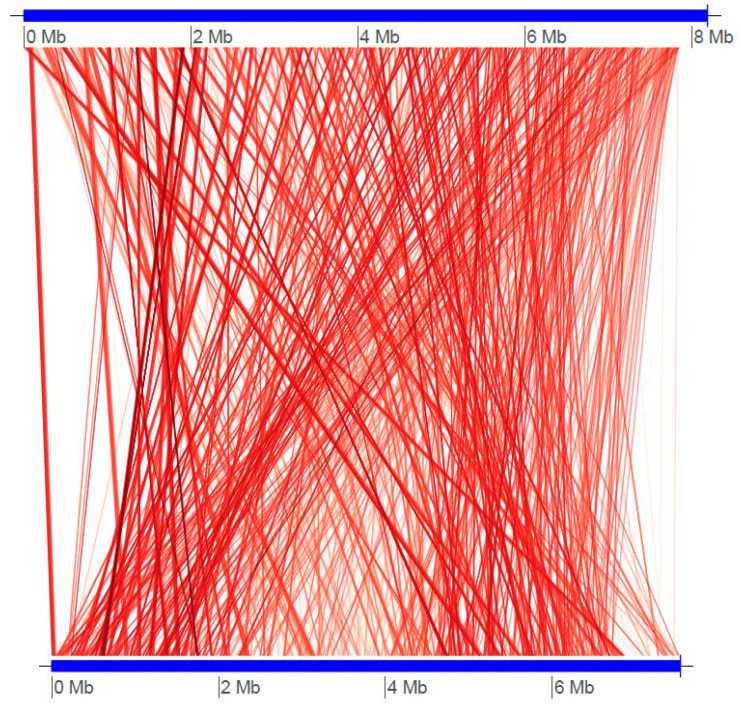
Genome alignment between *Streptomyces* isolate BR123 and *Streptomyces globosus*. Alignment was performed using the online KBase tool with default parameters. Synteny regions are represented by red lines, whereas breaks in synteny are the blank regions. Genome sizes are marked in the horizontal panels and conserved regions are linked.

The relationship with other species was verified by average nucleotide identity (ANI) scores, based on a previously used strategy [31,32]. The ANI value between *Streptomyces* sp. BR123 and *Streptomyces globosus* was found to be the maximum (87.3066) compared to the other *Streptomyces* species (Table 2) and the alignment between the two strains was strong (Figure 3).

### 3.2. Annotation and Assembly of Genome Sequence

**Figure 4:**
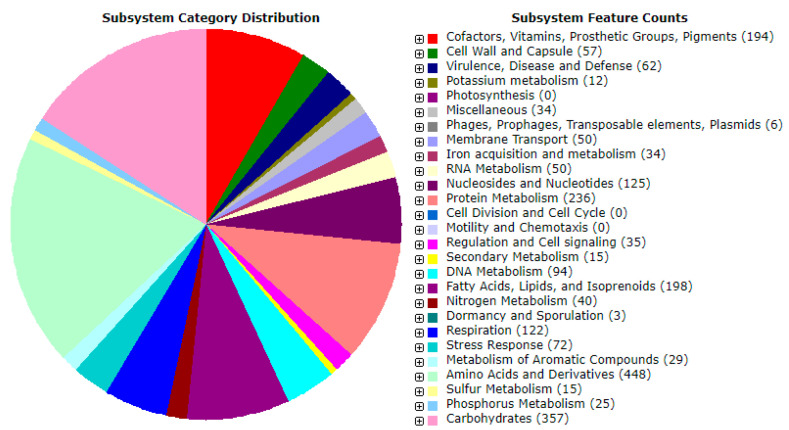
An overview of the subsystems for the genome of *Streptomyces* isolate BR123.

Automatic annotation performed by using the RAST server yielded 8038 features related to the protein coding genes. A total of 333 subsystems were identified using RAST genome analysis, which represented: the amino acid and derivative metabolism (448 ORFs); cofactors, vitamins, prosthetic groups, pigments (194 ORFs); and protein metabolism (236 ORFs). Ninety four open reading frames (ORFs) were involved in DNA metabolism, whereas 15 ORFs were found to code for secondary metabolites (Figure 4).

### 3.3. Biosynthetic Secondary Metabolite Gene Clusters of Streptomyces sp. BR123

About 70–80% of the total bioactive metabolites discovered so far relate to the genus *Streptomyces* [33]. Consequently, similar types of antimicrobial metabolites were found to be produced by *Streptomyces* strains, isolated from different environments [34]. Due to this de-duplication, rare actinobacteria have been targeted for the search of novel antimicrobial compounds [35]. The exploration of a genome-based biosynthetic potential of new isolates may be useful for finding novel compounds. In this study, a total of 44 clusters were identified in this strain, responsible for the production of secondary metabolites. This included 4 types of NRPS (nonribosomal peptide synthetase), 9 types of PKS (polyketide synthase), and 7 types of hybrid biosynthetic gene clusters. The hybrids featured melanin-terpene, lanthipeptide-3-NRPS, NRPS-transAT-PKS, T1 PKS-NRPS-like, T3 PKS-guanidinotides-RiPP-like, T1 PKS-NAPAA, and RRE-containing-thiopeptide. Most of the gene clusters detected in the isolate BR123 were related to polyketide biosynthesis. Out of the 44 biosynthetic gene clusters, 33 clusters represented differing percentages of resemblance with known BGCs, whereas 11 exhibited no similarity with known homologous gene clusters. The latter clusters were considered as orphan biosynthetic gene clusters [36] (Table 3). Particularly, the NRPS, NRPS-like, hybrid gene clusters, and majority of the peptide butyrolactone shared resemblance with antibacterial compounds, while most polyketides and other gene clusters shared similarity with anticancer and pigmented compounds. However, low degree of similarity was observed in most cases, suggesting the occurrence of possibly novel biosynthetic gene clusters [37,38]. 

The core structure of 15 clusters was predicted, which include 4 NRPS, 1 NRPS-like, 5 type I PKS, 1 PKS-like and 4 hybrid gene clusters. Moreover, a putative class II of lanthipeptide with a core peptide was also predicted (Supplementary File S1). Out of these clusters, 1 NRPS, 2 type-1 PKS, and the lanthipeptides were the orphan BGCs in *Streptomyces* sp. BR123 predicted by antiSMASH. The class II lanthipeptides are produced by the lanthionine synthase C (LanC) family protein that is present in cluster 59. Moreover, in the LanC enzyme of lanthipeptide class II, di-dehydroalanine (Dha) and di-dehydrobutyrine (Dhb) were well conserved.

Besides the core biosynthetic genes in *Streptomyces* isolate BR123, there were 10 clusters (clusters 9, 19, 24, 29, 40, 62, 89, 149, 183, 221) with transcription regulation and 8 clusters (clusters 11, 53, 76, 98, 157, 239, 279, 338) with transport genes, and there 7 clusters observed (clusters 3, 4, 16, 46, 59, 100, 104) with both transcription regulation and transport genes.

### 3.4. Detection of NRPS and PKS Genes in Streptomyces sp. BR123

The amplification and detection of NRPS and PKS genes via PCR further confirmed their presence in this *Streptomyces* strain (Supplementary Figure S3). *Streptomyces* sp. BR123 was also found to be active against a broad range of pathogenic microorganisms, including gram-positive and gram-negative bacteria and fungi. However, the activity was based on the media supplements used, and the maximum activity observed in the enrichment medium CSPY-ME resulted in the formation of the largest zone of inhibitions against some of the fungal and all of the tested bacterial strains. The maximum inhibitory effect was observed against *Bacillus* subtilis, showing a zone of inhibition with a diameter of 24.1 ± 0.12, followed by *E. coli* (23.5 ± 0.10) and *Aspergillus niger* (20.2 ± 0.08). No significant activities were observed in the ISP1 and ISP4 media (Supplementary Table S1), and the zone of inhibition in the ISP3 medium was only observed in Aspergillus niger (13.4 ± 0.05). Such a variation in activity could be due to different growth proportion in a minimal medium. Inhibition causes a greater effect in a minimal medium compared to a complex medium, where the medium’s ingredients may compensate for the inhibitory effect of the product formation [39].

### 3.5. Production of Secondary Metabolites by Streptomyces sp. BR123

**Figure 5:**
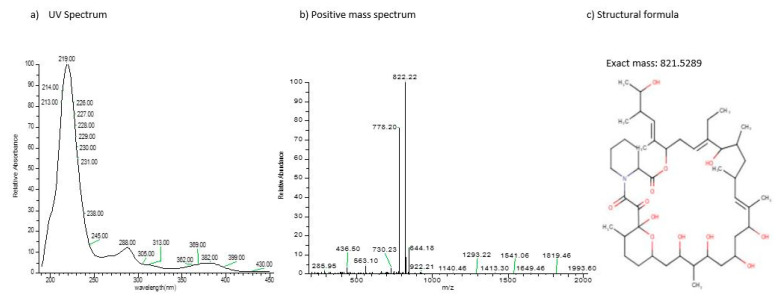
Characteristics of meridamycin, a metabolite observed from isolate BR123, calculated using HPLC-MS analysis. (**a**) The UV-visible spectrum; (**b**) the positive ion mass spectrum; and (**c**) the structural formula.

The production of various metabolites were verified through HPLC-MS [40,41,42]. A compound detected in the UV spectrum, with absorption maxima at 219 nm, 288 nm, and 369 nm, and a mass spectrum at positive ion mode with *m*/*z* ratio of 822.22 was identified as meridamycin, with a molecular mass of 821.5 (Figure 5). 

**Figure 6:**
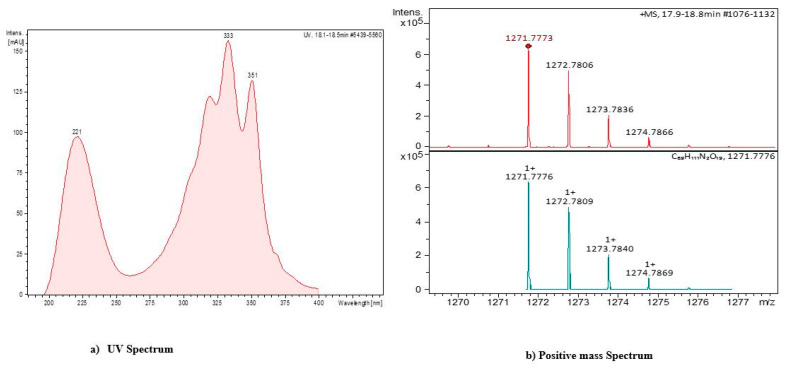
Characteristics of unidentified metabolite from the *Streptomyces* isolate BR123 based on (**a**) UV spectrum; (**b**) HPLC-MS analysis.

Meridamycin is a macrocyclic polyketide which possesses non-immunosuppressive, neuroprotective activity by acting on dopaminergic receptors and has been found to be suitable for the treatment of neurological diseases [43]. A small number of studies have reported the production of this compound from the genus *Streptomyces* during the last few years [43,44], and evidence on the presence of the biosynthetic pathway of this compound in *Streptomyces* sp. DSM 4137 has been published [44]. Moreover, various therapeutically important metabolites analogous to meridamycin have also been previously identified [45]. Another compound with absorbance maxima at 221 nm, 333 nm, and 351 nm and a molecular mass of 1271 at positive ion mode (Figure 6) was also observed. Upon library screening, it was observed to not correspond with any known compound, thus further characterization is required. The compound analysis of *Streptomyces* sp. BR123 indicated the potential of this strain as a candidate for the production of novel secondary metabolites.

## 4. Conclusions

Due to the development of multi-drug resistance (MDR) by emerging pathogens against the available antibiotics, there is a dire need to find new sources of antibiotics. The genus *Streptomyces* has massively contributed to the field of medicine through the synthesis of antibacterial, antifungal, antiparasitic, and anticancerous compounds. In the current study, we explored an indigenously isolated potent bioactive *Streptomyces* strain, and added another draft genome sequence to the rising number of *Streptomyces* sequences in the repository. Moreover, a few already known compounds in addition to some new and uncharacterized compounds were also detected using the HPLC-MS technique. This genome insight study of *Streptomyces* sp. BR123 and the information about the biosynthetic clusters of some uncharacterized natural compounds may prove to be a valuable addition to prior knowledge, assisting in the search for novel compounds as well as providing the much-needed structural diversity required for a new generation of antibiotics designed for pathogens with MDR. 

## Figures and Tables

**Table 1:** General genomic features of *Streptomyces* sp. isolate BR123 and other species used in this study.

Strain	Bio-Project Accession	Size (Mbps)	No. of Contigs	% G + C	CDS	tRNA	rRNA
*Streptomyces* sp. isolate BR123	PRJNA643667	8.16	723	72.63	8103	68	8
*Streptomyces globosus* LZH-48	PRJNA428275	7.54	-	73.62	6524	71	3
*Streptomyces katrae* NRRL ISP-5550	PRJNA238534	8.05	1874	72.69	7305	56	2
*Streptomyces virginiae* NRRL ISP-5094	PRJNA238534	8.32	133	72.4	7245	74	13
*Streptomyces clavuligerus* F1D7	PRJNA679926	7.59	-	72.5	6122	65	18
*Streptomyces diastaticus* NBRC 15402	PRJDB6184	7.85	32	72.7	-	75	8
*Streptomyces bacillaris* ATCC 15855	PRJNA471017	7.89	-	72.0	6746	65	18
*Streptomyces cyaneofuscatus* SID 10855	PRJNA603111	7.88	52	71.6	6755	66	12
*Streptomyces griseus* NBRC 13350	PRJDA20085	8.55	-	72.2	7087	67	18
*Streptomyces lavendulae* YAKB-15	PRJNA526603	7.77	100	72.2	7009	70	21

**Table 2:** Average nucleotide identity (ANI) between all *Streptomyces* species used in this study.

Query	Reference	ANI Estimate	Matches	Total
*Streptomyces lavendulae* subsp. *lavendulae*	*Streptomyces* sp. isolate BR123	81.6134	1070	2300
*Streptomyces* sp. isolate BR123	*Streptomyces lavendulae* subsp. *lavendulae*	81.673	1050	2391
*Streptomyces* sp. isolate BR123	*Streptomyces virginiae*	86.0723	1576	2391
*Streptomyces virginiae*	*Streptomyces* sp. isolate BR123	86.0802	1554	2721
*Streptomyces globosus*	*Streptomyces* sp. isolate BR123	87.1686	1630	2510
*Streptomyces* sp. isolate BR123	*Streptomyces globosus*	87.3066	1626	2391
*Streptomyces* sp. isolate BR123	*Streptomyces katrae*	87.1854	1671	2391
*Streptomyces katrae*	*Streptomyces* sp. isolate BR123	87.2335	1635	2813

**Table 3:** List of putative secondary metabolites producing biosynthetic gene clusters as predicted by antiSMASH.

Cluster	Size (bp)	Most Similar Known Biosynthetic Gene Cluster	MIBG BGC-ID
**Siderophores:**			
3	11,590	-	-
56	6349	-	-
226	8264	Desferrioxamin B (100%)	BGC0000941
261	8036	Ficellomycin (7%)	BGC0001593
279	6963	Ficellomycin (7%)	BGC0001593
**Terpenes:**			
9	16,885	-	-
11	21,676	Hopene (61%)	BGC0000663
16	21,086	-	-
19	13,165	-	-
24	25,408	Isorenieratene (63%)	BGC0001227
69	13,506	Ebelactone (5%)	BGC0001580
**PKS:**			
2 (Type I)	103,249	Concanamycin A (21%)	BGC0000040
4 (Type I)	46,281	Clifednamide A (30%)	BGC0001553
94 (Type I)	23,404	Tetrocarcin A (8%)	BGC0000162
129 (Type I)	19,401	-	-
320(Type I)	7593	-	-
350(Type I)	6899	-	-
58 (Type II)	34,290	Granaticin (16%)	BGC0000227
89 (Type III)	24,296	Alkylresorcinol (100%)	BGC0000282
338 (Type III)	7187	Flaviolin (75%)	BGC0000902
**NRPS:**			
104	23,007	Lactonamycin (5%)	BGC0000238
271	9618	Griseoviridin/Fijimycin A (8%)	BGC0000459
239	11,133	-	-
401	5437	Virginiamycin S1 (11%)	BGC0001116
**Peptides:**			
59 (Lanthipeptide class II)	13,149	-	-
76 (Lanthipeptide class I)	23,247	Chejuenolide A/Chejuenolide B (7%)	BGC0001543
**Butyrolactones:**			
100	6302	Griseoviridin/Fijimycin A (8%)	BGC0000459
**NRPS/PKS-like:**			
221 (NRPS-like)	12,004	Lipstatin (14%)	BGC0000382
429 (NRPS-like)	4493	Glycinocin A (4%)	BGC0000379
243 (PKS-like)	10,893	Virginiamycin S1 (33%)	BGC0001116
**Hybrids:**			
3 (Melanin, terpene)	33,435	Melanin (40%)	BGC0000909
29 (Lanthipeptide-3, NRPS)	43,146	Azicemicin (8%)	BGC0000202
46 (NRPS, transAT-PKS)	36,866	Virgimiamycin S1 (55%)	BGC0001116
62 (Type I PKS, NRPS-like)	29,119	Monensin (26%)	BGC0000100
98 (Type III PKS, guanidinotides, RiPP-like)	23,202	Pheganomycin (52%)	BGC0001148
149 (Type I PKS, NAPAA)			
433 (RRE-containing, thiopeptide)	17,747	Mediomycin A (34%)	BGC0001661
	4312	Lactazol (33%)	BGC0000606

## Data Availability

https://www.ncbi.nlm.nih.gov/assembly/GCF_013401435.1/ (10 July 2020).

## Supplementary Materials

The following supporting information can be downloaded at https://www.mdpi.com/article/10.3390/antibiotics11081057/s1, Figure S1: A plot representing the number of contigs of the *Streptomyces* sp. BR123 genome with the GC percentage in a certain range; Figure S2: The phylogenetic tree of *Streptomyces* isolate BR123 and other *Streptomyces* species based on 16S rRNA sequences; Figure S3: PCR-based identification of NRPS and PKS genes in isolate BR123. (a) NRPS (b) PKS-I (c) PKS-II; File S1: Biosynthetic gene clusters predicted by antiSMASH and their core structures; Table S1: Antimicrobial activity of *Streptomyces* strain BR123 in different growth media.
