# Supplementary material for: Revealing Genome-Based Biosynthetic Potential of Streptomyces sp. BR123 Isolated from Sunflower Rhizosphere with Broad Spectrum Antimicrobial Activity
Source: Antibiotics (Basel). 2022 Aug 4;11(8):1057. doi: 10.3390/antibiotics11081057 (PMC9405382; doi:10.3390/antibiotics11081057)
Supplement: Supplementary file 1 [file antibiotics-11-01057-s001.zip › antibiotics-1823231-supplementary.pdf]

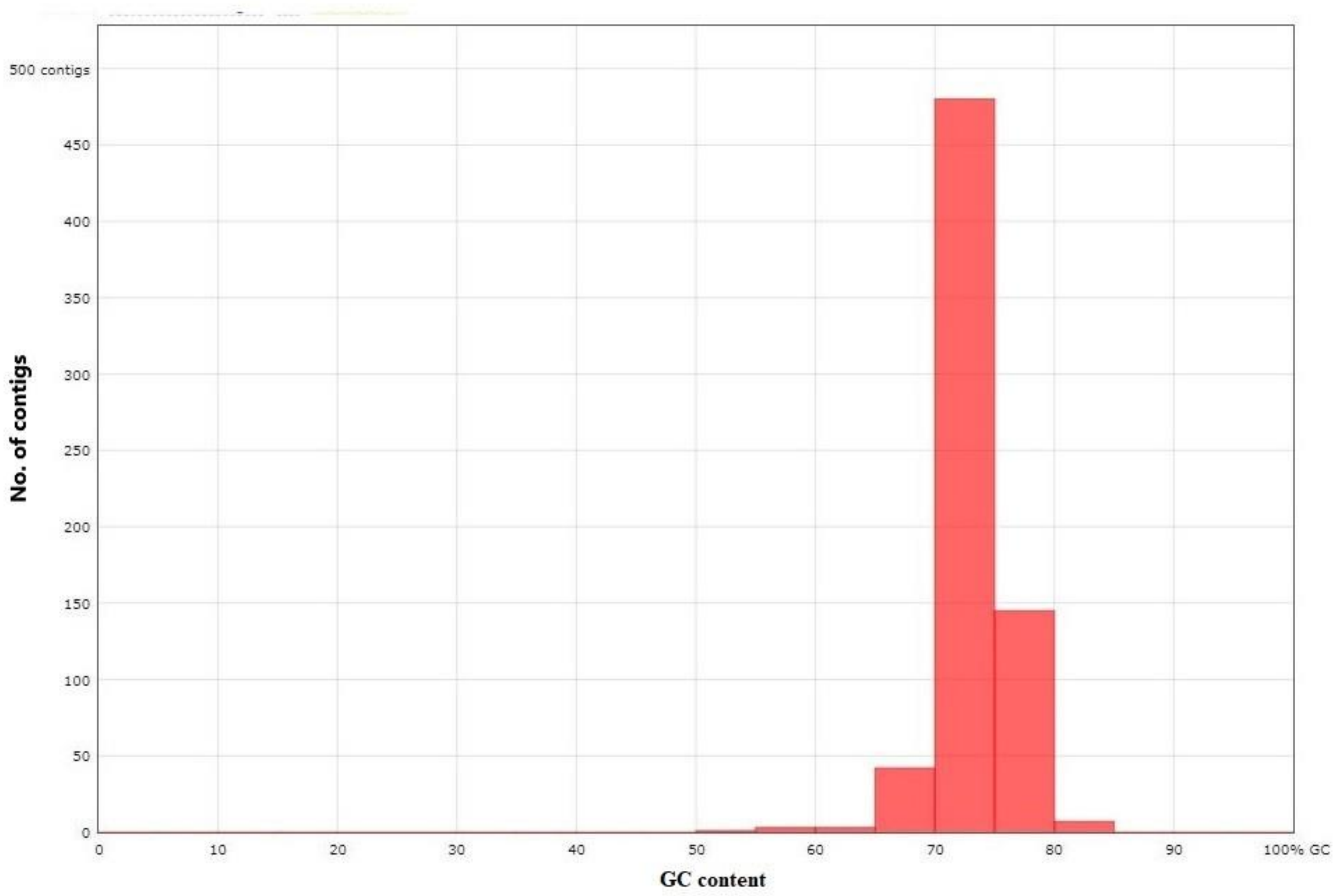

Figure S1. Plot representing number of contigs of the *Streptomyces* sp. BR123 genome with GC percentage in certain range

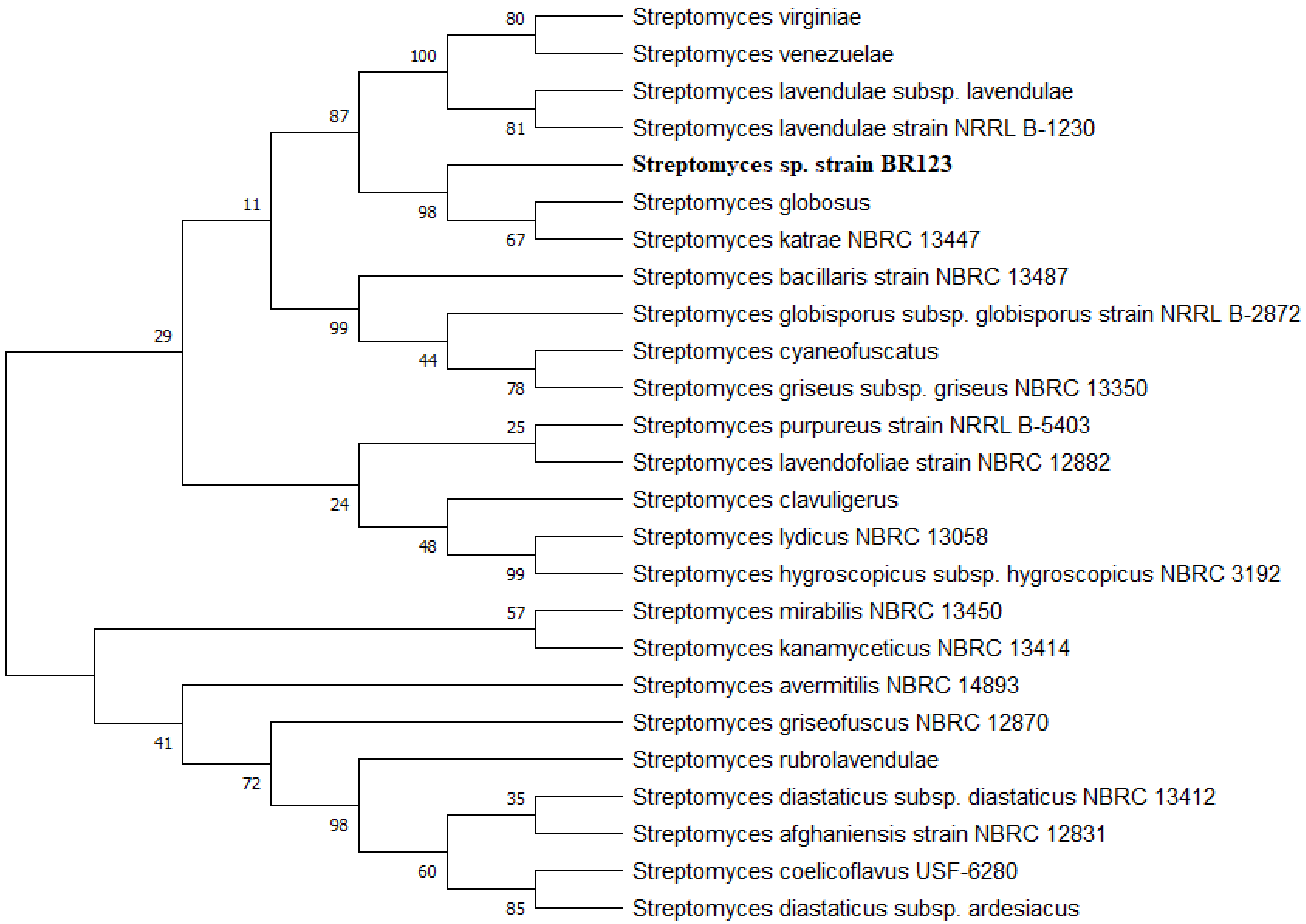

Figure S2. Phylogenetic tree of *Streptomyces* isolate BR123 and other *Streptomyces* based on 16S rRNA sequences

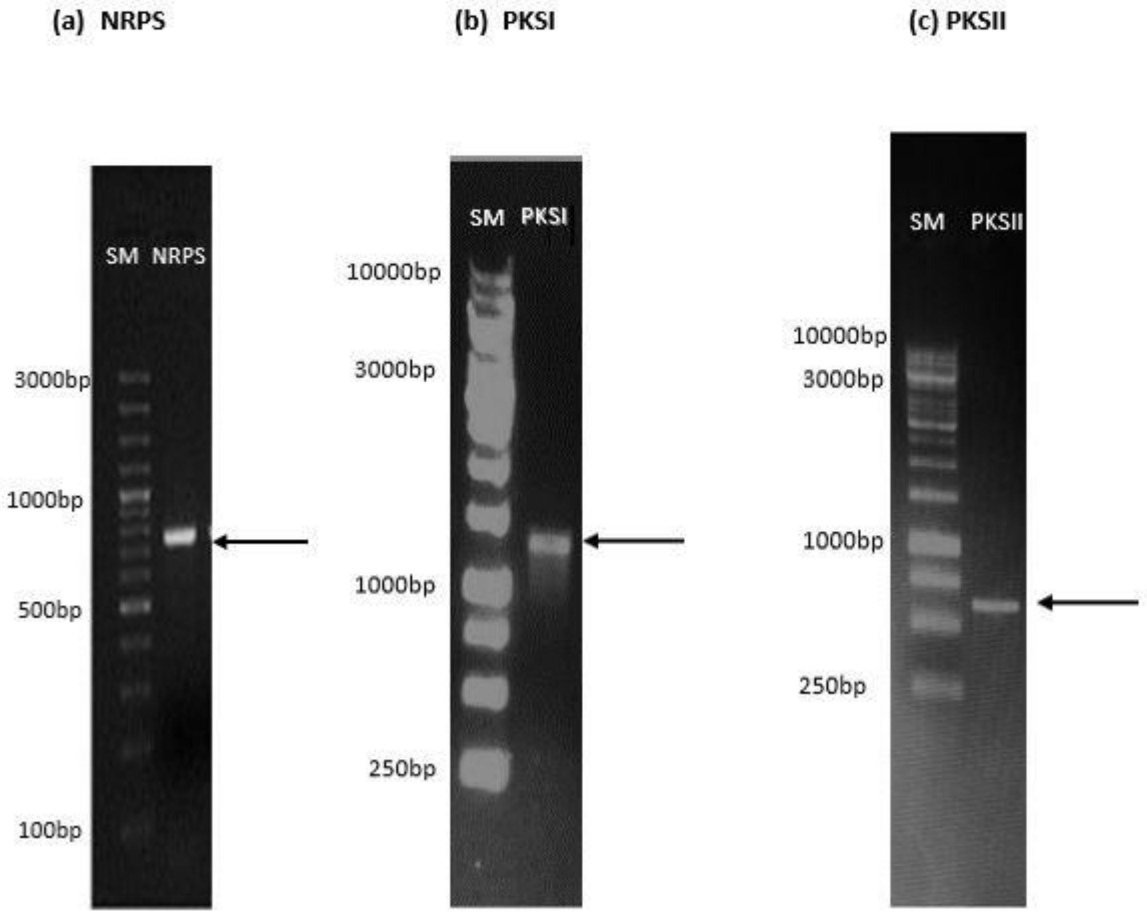

Figure S3. PCR-based identification of NRPS and PKS genes in isolate BR123.cite (a) NRPS (b) PKS 1 (c) PKS 2

File S1. Biosynthetic gene clusters predicted by antiSMASH cite and their core structures.

(a) Cluster 59: Lanthipeptide class-ii

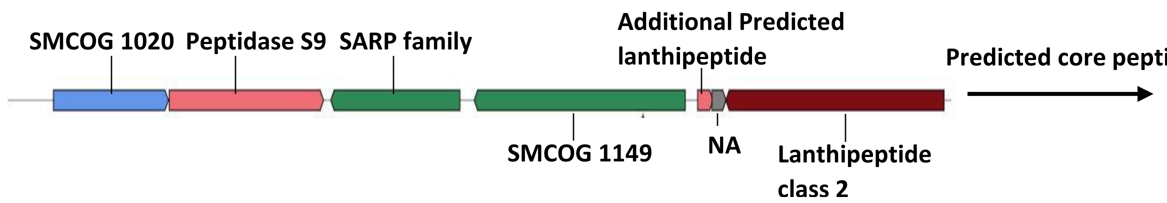

MGMSGKAAALASADLDhaDhaRLRDhaPDhaIPIAPNDhbIDhaPDhbGWDhaWAAADhaAPAPLDhaDhbYLDhbEDhaLICMVIRPDhaYPQVWWDhaC
(Dha: Didehydroalanine, Dhb: Didehydrobutyrine)

(b) Cluster 2: T1PKS

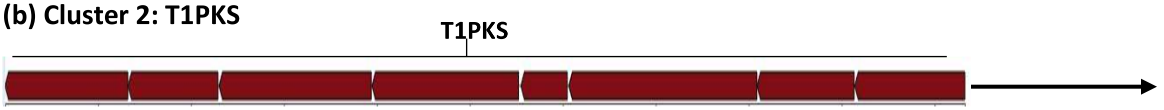

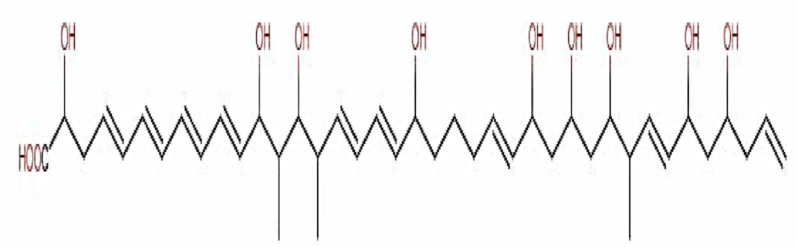

(c) Cluster 4: T1PKS

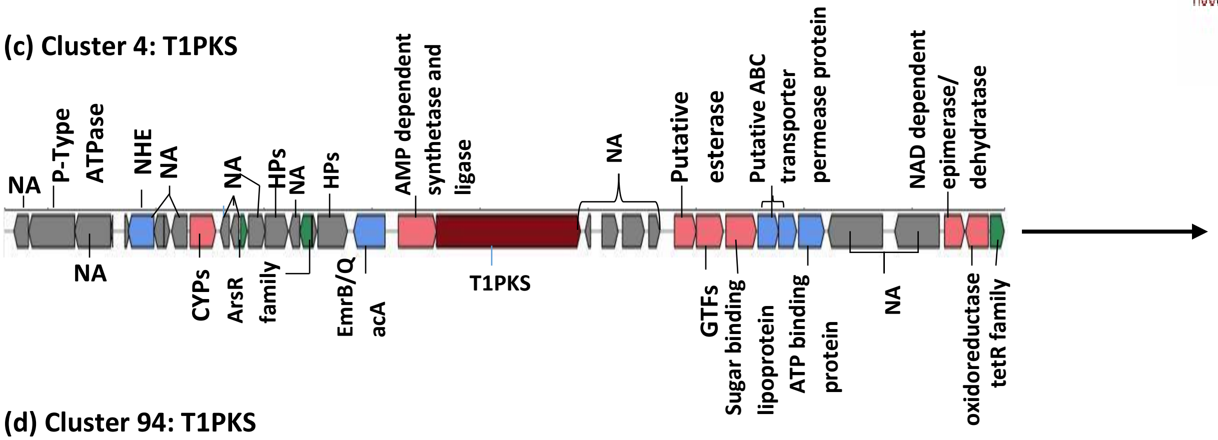

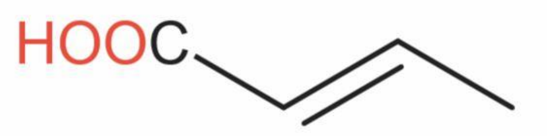

(d) Cluster 94: T1PKS

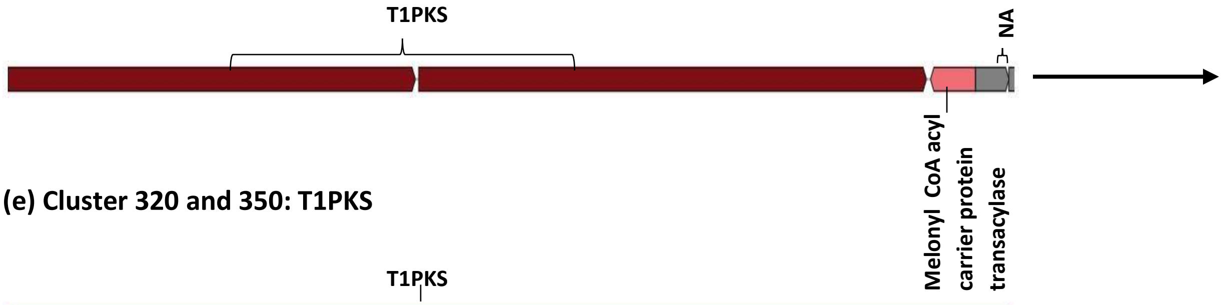

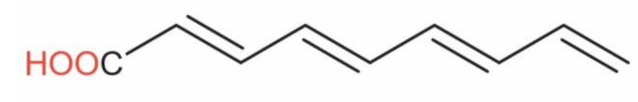

(e) Cluster 320 and 350: T1PKS

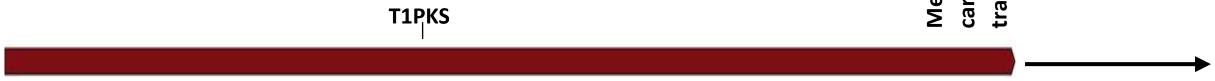

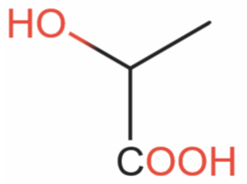

(f) Cluster 104: NRPS

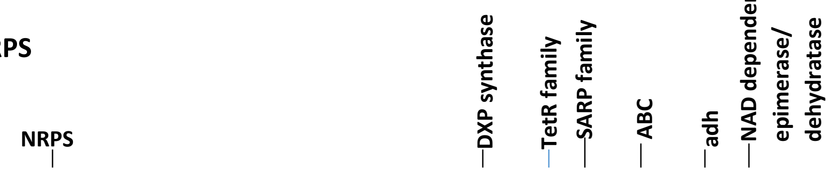

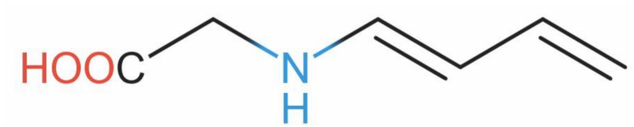

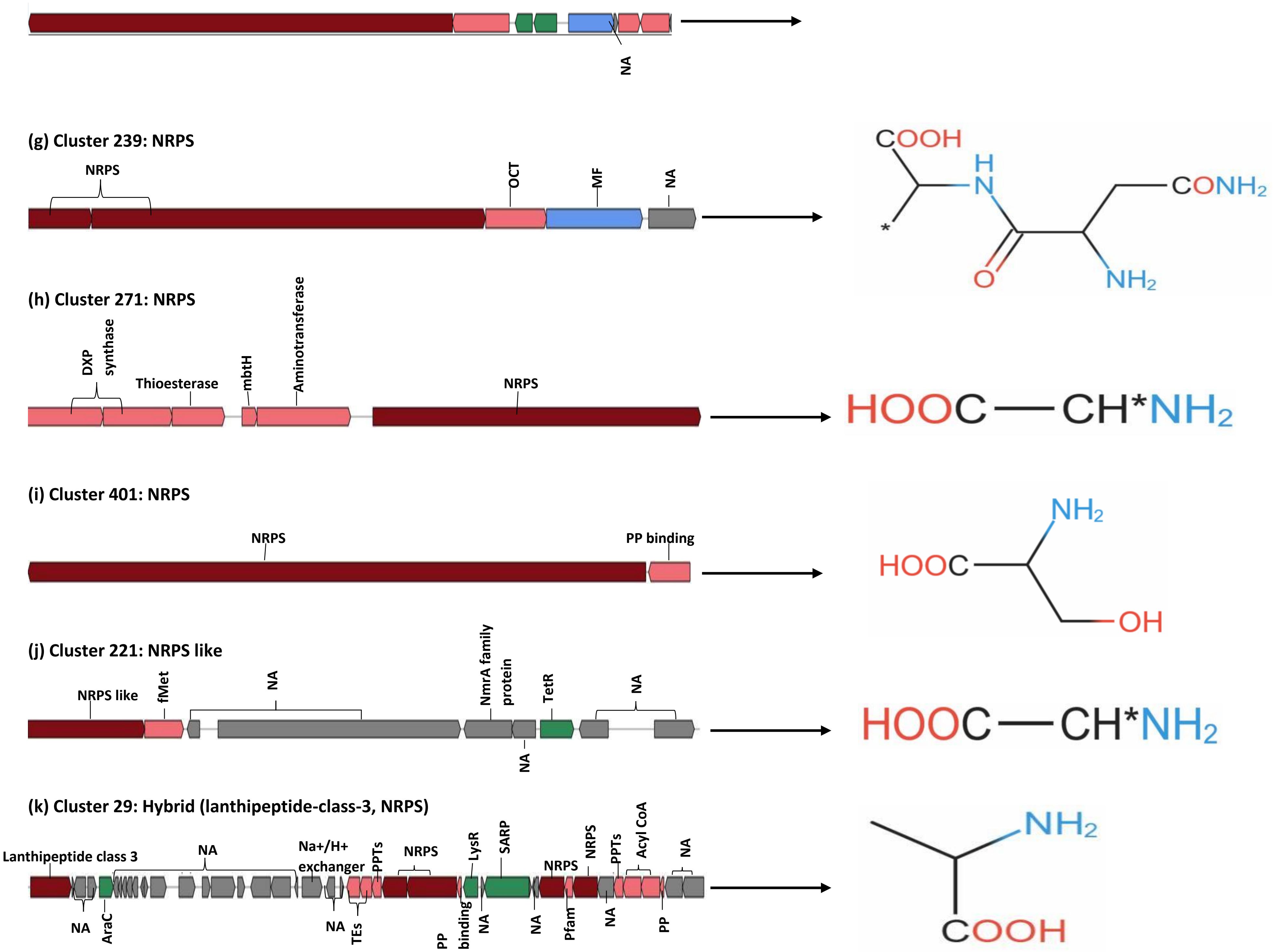
NA
(g) Cluster 239: NRPS
NRPS
OCT
MF
NA
COOH
H
N
CONH2
*
O
NH2
(h) Cluster 271: NRPS
DXP synthase
Thioesterase
mbtH
Aminotransferase
NRPS
HOOC—CH*NH2
(i) Cluster 401: NRPS
NRPS
PP binding
NH2
HOOC
OH
(j) Cluster 221: NRPS like
NRPS like
fMet
NA
NmrA family protein
TetR
NA
NA
HOOC—CH*NH2
(k) Cluster 29: Hybrid (lanthipeptide-class-3, NRPS)
Lanthipeptide class 3
NA
Na+/H+ exchanger
PPTs
NRPS
LysR
SARP
NRPS
NRPS
PPTs
Acyl CoA
NA
NA
AraC
NA
TEs
PP binding
NA
NA
Pfam
NA
PP
NH2
COOH

(l) Cluster 46: Hybrid (NRPS, transAT-PKS)

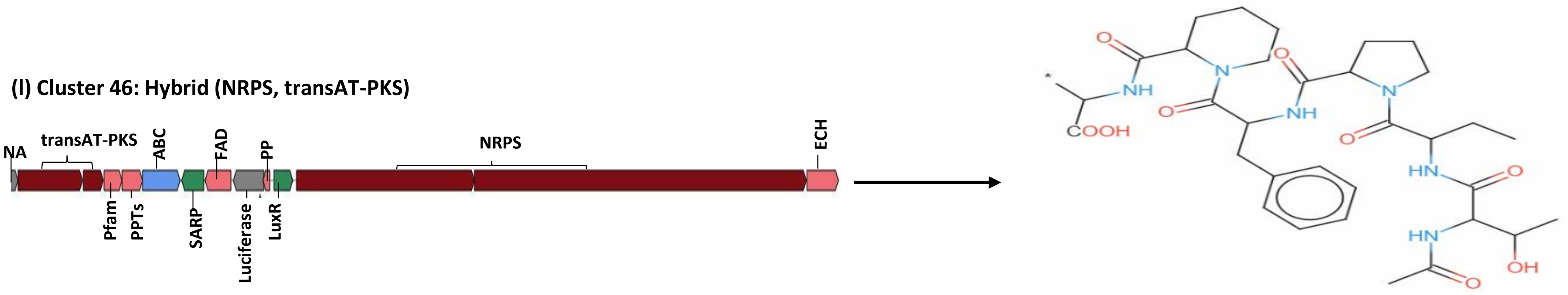

(m) Cluster 62: Hybrid (T1PKS, NRPS like)

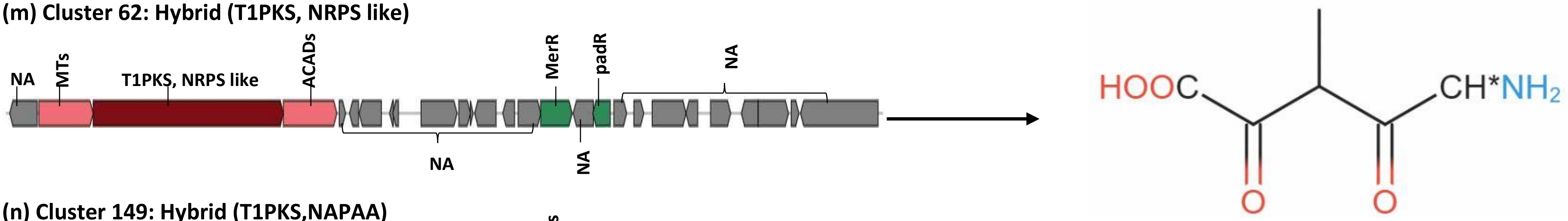

(n) Cluster 149: Hybrid (T1PKS,NAPAA)

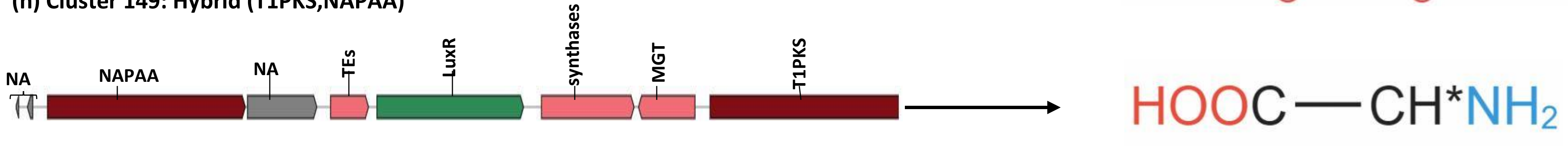

(o) Cluster 243: PKS like

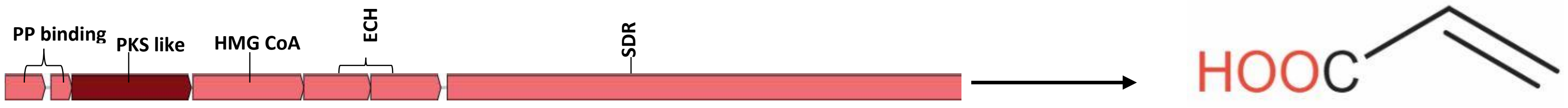

Table S1. Antimicrobial activity of *Streptomyces* strain BR123 in different growth media

Test organisms	Culture Medium*(Zone of inhibition in mm)						
	ISP1	ISP2	ISP3	ISP4	ISP5	SCA	CSPY-ME
**Gram-positive bacteria**							
Staphylococcus aureus	-	-	-	-	13.2±0.19	-	16.4±0.09
Bacillus subtilis	-	-	-	-	-	-	24.1±0.12
**Gram-negative bacteria**							
Salmonella typhi	-	-	-	-	-	-	11.6±0.11
Xanthomonas oryzae	-	21.7±0.11	-	-	-	-	18.4±0.02
Escherichia coli	-	12.4±0.23	-	-	20.3±0.08	16.6±0.18	23.5±0.10
Pseudomonas aeruginosa	-	-	-	-	-	-	8.9±0.14
**Fungus**							
Aspergillus niger	-	18.2±0.12	13.4±0.05	-	10.9±0.24	-	20.2±0.08
Aspergillus flavus	-	14.3±0.33	-	-	12.5±0.17	-	-
Fusarium oxysporum	-	-	-	-	-	-	-
Fusarium solani	-	17.5±0.25	-	-	15.1±0.07	-	18.7±0.21

*ISP (International *Streptomyces* project) medium including; ISP1, trypton yeast extract agar (276910/ BD Difco/ fisher scientific, USA); ISP2, Yeast extract malt extract agar (277010/ BD Difco/ fisher scientific, USA); 2; ISP3, Oat meal agar (O 3506/SIGMA, USA); ISP4, inorganic salt starch agar (277210/ BD Difco/ fisher scientific, USA); ISP5, glycerol asparagine agar (G9788/SIGMA-ALDRICH, USA);
SCA, starch casein agar (Section Materials and Methods);
CSPY-ME, casein-starch-peptone-yeast extract-malt extract agar (Section Materials and Methods)
